# *TP53* Pro72 Allele Is Enriched in Oral Tongue Cancer and Frequently Mutated in Esophageal Cancer in India

**DOI:** 10.1371/journal.pone.0114002

**Published:** 2014-12-01

**Authors:** Raju S. R. Adduri, Rajender Katamoni, Ramaswamy Pandilla, Sandeep N. Madana, Arun Kumar Paripati, Viswakalyan Kotapalli, Murali Dharan Bashyam

**Affiliations:** Laboratory of Molecular Oncology, Centre for DNA Fingerprinting and Diagnostics, Nampally, Hyderabad, India; The University of Hong Kong, China

## Abstract

**Purpose:**

The tumor suppressor p53 is known to be inactivated frequently in various cancers. In addition, germline polymorphisms in *TP53* are known to affect protein function and influence risk of developing different types of cancers. In this study, we analyzed the association of *TP53* Pro72Arg polymorphism with squamous cell carcinoma of oral tongue (SCCOT) and esophagus (ESCC) in India.

**Methods:**

We assessed the distribution of *TP53* Pro72Arg polymorphism in one hundred and fifteen and eighty two SCCOT and ESCC patients, respectively, with respect to one hundred and ten healthy controls from the same population. In addition, we analyzed association of the polymorphism with several clinico-pathological and molecular parameters.

**Results:**

Pro72 allele was significantly enriched in SCCOT patients compared to the healthy control group but neither allele was enriched in ESCC. Interestingly, Pro72 allele was preferentially mutated in ESCC which was confirmed by analysis of samples heterozygous for Pro72Arg.

**Conclusions:**

Our study revealed the association of Pro72 allele with SCCOT suggesting the effect of this polymorphism on SCCOT risk. Preferential mutation of Pro72 allele exclusively in ESCC indicates the need for further studies to understand the tissue specific effect of p53 polymorphism.

## Introduction

Squamous Cell Carcinoma of the Oral Tongue (SCCOT) is a common form of head and neck squamous cell carcinoma (HNSCC) and its incidence is consistently increasing worldwide [1]. The increase in incidence has been noted predominantly in younger patients [2], [3] in whom it appears to exhibit lower association with common risk factors such as alcohol and tobacco suggesting possible genetic susceptibility [3]. SCCOT is known to be aggressive and is associated with higher rates of occult and nodal metastasis when compared to other HNSCC subtypes [4]. It is often associated with poor survival which has not improved significantly over the past four decades [5], [6]. Esophageal cancer (EC) is the sixth and eighth most common cancer in males and females respectively in India (http://www.icmr.nic.in/ncrp/report_pop_2001-04/cancer_p_based.htm). Squamous (ESCC; occurs usually in proximal and middle esophagus) and adeno (EAC; occurs mainly in distal esophagus) carcinoma are the two major EC subtypes [7]. Though ESCC was more common few decades ago, a rapid increase in EAC has been noted since the 1980s in the West [8], in parallel with an increased incidence of gastrointestinal reflex disease (GERD). GERD causes an inflammation induced pathological condition called ‘Barrett's esophagus’, which predisposes to EAC [9]. Though, a similar trend in increase of GERD has been noted in Asian countries in the last few decades, ESCC remains the predominant EC subtype [10], suggesting possibility of role of genetic factors. Due to its closer location to neck and similarities in tumorigenesis pathways, ESCC is sometimes classified with HNSCC.

Somatic inactivation of *TP53* is a frequent event in most cancers whereas germ line aberrations are associated with Li-Fraumeni [11], a hereditary cancer predisposition syndrome. Common modes of p53 inactivation are point mutations, allelic loss [12] and inactivation mediated by oncoviral proteins [13]. In addition, numerous polymorphisms occur in *TP53* of which a few are suggested to perturb protein function and may influence cancer susceptibility [14]. Among these, the codon 72 Pro/Arg polymorphism (rs1042522) is the most common and well-studied. The two p53 codon72 alleles encode proline (Pro72) or arginine (Arg72), located in a polyproline region present between the transactivation and the DNA binding domains and may affect the structure of the putative SH3-binding domain [15]. The Pro72 allele is known to be associated with coronary artery disease [16], higher susceptibility to endometriosis [17], primary open angle glaucoma [18], systemic lupus erythematosus (especially in Asians) [19] and ulcerative colitis [20] whereas the Arg72 allele is associated with progression of Diabetic Nephropathy [21]. More importantly, the polymorphism exhibits varying association with risk [22], [23], survival [24], [25], and treatment response [25] in several cancers in different populations.

In the current study, we assessed the frequency of Pro72Arg polymorphism in SCCOT and ESCC. Pro72 allele appeared to be significantly associated with SCCOT whereas no association with either allele was detected in ESCC. However, in ESCC, *TP53* DNA binding domain mutations occurred at a significantly higher frequency in the Pro72 allele.

## Materials and Methods

### Patient and control samples

One hundred and fifteen and eighty two previously untreated and surgically resected SCCOT and ESCC samples respectively were collected during the period 2007 to 2013 from three hospitals in Hyderabad, India, following informed consent. Seventy two and seventy five SCCOT and ESCC patients respectively were from our earlier studies [26], [27]. Clinico-pathological details of the patients are given in Table S1. Median age and male to female ratio were 49 and 50; and 1.94 and 1 for SCCOT and ESCC respectively. Peripheral blood from one hundred and ten age and gender matched cancer free healthy individuals belonging to the same geographical region were collected.

### Ethics statement

The study was approved by ethics committees of Apollo Hospitals (14/05/2005), MNJ Institute of Oncology and Regional Cancer Centre (23/09/2006 and 20/10/2008) and Indo-American Cancer Institute and Research Centre (08/08/2007) as well as Institutional bioethics committee of Centre for DNA Fingerprinting and Diagnostics (CDFD) (20/12/2009) as per modified Helsinki declaration 2005. All samples were collected following written informed consent.

### Genotyping

**Figure 1:**
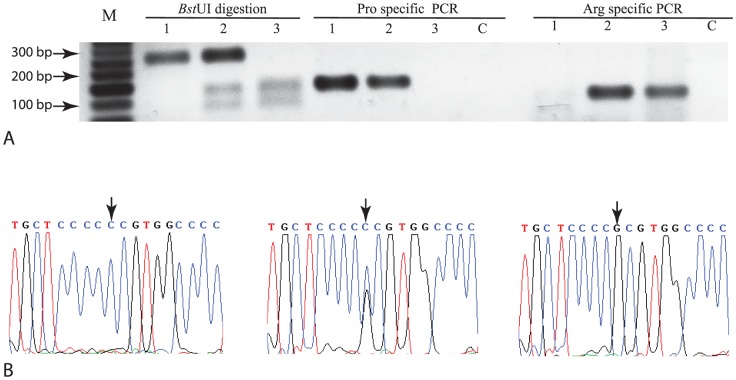
Identification of the three different p53 codon 72 genotypes. Panel A, PCR-RFLP analysis confirmed by allele specific PCR performed as described in the materials and methods section. M, 50 bp DNA ladder; 1, Pro/Pro; 2, Pro/Arg; 3, Arg/Arg; and C, No template control. Panel B, Sanger sequencing. Pro/Pro (left), Pro/Arg (middle) and Arg/Arg (right).

DNA was isolated from histologically confirmed normal tissue adjacent to tumor for each sample as detailed in Document S1. Genotyping of codon 72 was performed using a two-pronged approach including PCR-RFLP (Figure 1A) [28] and allele specific PCR (Figure 1A) [29] as previously described. The results were independently confirmed in twenty and fifteen SCCOT and ESCC samples respectively using bidirectional Sanger sequencing on a 3100 Genetic analyzer (ABI Inc., Foster city, CA, USA) (Figure 1B). Primer sequences are listed in Table S2.

### *TP53* mutation screening

*TP53* mutation screening was performed as described earlier [26]. To identify whether the Pro72 or the Arg72 allele was harboring p53 mutation in samples heterozygous for the Pro72Arg polymorphism, a long amplicon (∼2700 bp) spanning exons 4–8 of *TP53* (which includes the codon 72 as well as the region encoding the DNA binding domain) was amplified (Primer pairs Arg^+^ and Exon 8R, Table S2) from genomic DNA and cloned into TA vector (Invitrogen, Carlsbad, CA, USA). The recombinant plasmids were screened for mutation as well as for the codon 72 polymorphism using bidirectional Sanger sequencing.

### Molecular analysis

Status of p53 nuclear stabilization, *TP53* mutation (only for SCCOT), EGFR over expression, microsatellite instability, β-catenin nuclear localization status (only for ESCC), HPV infection and LOH and clinico-pathological variables including age, gender, smoking, alcohol consumption, grade and pathological stage were reported in our previous studies [26], [27].

### Statistical analysis

Deviation from Hardy-Weinberg equilibrium for genotype frequency for cases and controls, was analyzed using χ^2^ test. Odds ratio and corresponding 95% confidence intervals for disease risk were calculated. Dominant, codominant, recessive, over dominant and log additive inheritance models were tested to determine whether the SNP was associated with disease. Akaike (AIC) and Bayesian (BIC) information criterion in addition to χ^2^ p values were used to select the best model of inheritance. Association between different genotypes and clinico-pathological variables was assessed using χ^2^ or Fisher exact test as appropriate.

## Results

### *TP53* Pro72 allele is enriched in SCCOT but not in ESCC patients

We analyzed the distribution of p53 codon 72 genotypes in SCCOT and ESCC with respect to healthy controls (Figure 1) as described in materials and methods section. 26 (23.6%), 53 (48.2%) and 31 (28.2%) control samples harbored Pro/Pro, Pro/Arg and Arg/Arg genotypes, respectively, exhibiting thereby no significant enrichment of one allele over the other (Table 1). However, Pro72 allele appeared to be significantly enriched in SCCOT compared to controls (Table 1) whereas no significant enrichment was observed in ESCC (Table 1). The genotype distributions of SCCOT and ESCC samples as well as controls were not deviating from Hardy-Weinberg equilibrium (Table 1). Co-dominant, recessive and log-additive genetic models were found to be appropriate for the inheritance of SCCOT for this SNP (Table S3A). However, lowest value of AIC (310.7) and BIC (317) for recessive model indicates it to be the best model (Table S3A). In contrast, ESCC did not exhibit association with any genetic model (Table S3B) as expected. None of several molecular and clinico-pathological variables exhibited significant association with codon 72 allele frequency in SCCOT and ESCC (Table S1).

**Table 1:** Comparative analysis of p53 Pro72Arg polymorphism status in SCCOT, ESCC and healthy control samples.

	Genotype distribution^a^				Allele distribution		
	Pro/Pro	Pro/Arg	Arg/Arg	P^c^	Pro72	Arg72	P^c^
**Controls (110)** b	26 (23.6%)	53 (48.2%)	31 (28.2%)	-	105 (47.7%)	115 (52.3%)	-
**SCCOT (115)** b	44 (38.3%)	48 (41.7%)	23 (20%)	0.051	136 (59.1%)	94 (40.9%)	0.019
**ESCC (82)** b	20 (24.4%)	46 (56.1%)	16 (19.5%)	0.363	86 (52.4%)	78 (47.6%)	0.361

a χ^2^ test p values for deviation from Hardy-Weinberg equilibrium are 0.71, 0.18 and 0.37 for controls, SCCOT and ESCC, respectively.

b Number in parenthesis indicates total number of samples.

c p is from χ^2^ test performed for comparison of SCCOT or ESCC with controls.

### *TP53* mutations are exclusively observed in ESCC tumors with p53 nuclear stabilization

*TP53* mutation status was reported earlier for all SCCOT samples analyzed in the previous study [26]; mutations were associated with poor disease specific survival [26] but not with several clinico-pathological parameters enumerated in materials and methods section. We screened ESCC samples for somatic mutations in exons 5–8 of *TP53* that encode the DNA binding domain and are known to harbor majority of cancer associated mutations [30]. Mutations were detected in twenty nine of forty five samples exhibiting nuclear stabilization and none of thirty seven samples that did not, suggesting that absence of nuclear stabilization may be a reliable indicator of absence of *TP53* DNA binding domain mutation in ESCC. A total of twenty seven (nineteen missense, three nonsense and five indels) mutations (all heterozygous) were identified. Two mutations (c.610G>T (p.E204X) and c.566delC (p. P190Lfs*57)) were detected in two samples each (Table S4). We identified three novel somatic mutations *viz.*, c.621_639del19, c.454_466dupCCGCCCGGCACCC and c.428_432delTGCAG+ 440delG (Table S4). Detailed description of the novel mutations is given in Document S2. Mutations in ESCC were not associated with any of the clinico-pathological variables analyzed (Table S5). Proportion of transitions and transversions (Figure S1A) as well as frameshift, missense and nonsense mutations (Figure S1B) were similar to previous reports for ESCC as per the International Agency for Research on Cancer (IARC) *TP53* database [15]. Interestingly, proportion of deletions was higher than reported in the *TP53* database (Figure S1B). G:C>A:T transitions constituted the major *TP53* mutation type (9/29; 34.48%) in ESCC samples in this study similar to the database [15]. However, frequency of C>T transitions at CpG dinucleotides was lower (10.34%) while frequency of deletions (5/29; 17.24%) was higher than reported in the database [15].

### Pro72 allele harbored inactivating mutations frequently in ESCC

**Figure 2:**
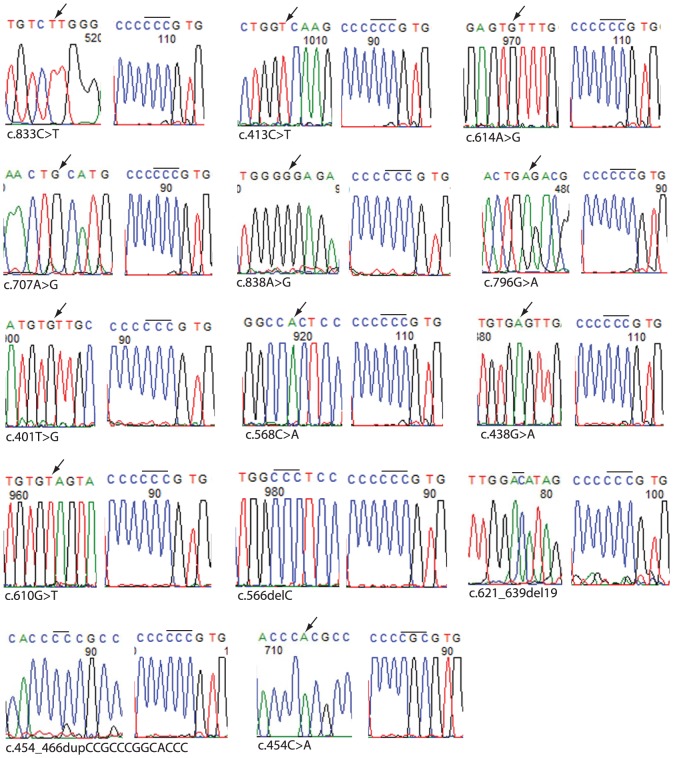
*TP53* mutation identified in Pro72 or Arg72 allele in fourteen ESCC samples heterozygous for p53 codon 72 polymorphism. Electrophoretograms showing mutation (left) and codon 72 polymorphism (right) for each of the fourteen samples are shown. Location of missense and deletion mutations are indicated by arrows and bars, respectively. Proline (CCC) or Arginine (CGC) codons are indicated by a bar.

P53 DNA binding mutations were not associated with codon 72 genotype in SCCOT (p = 0.917) (Table 2). In contrast, ESCC samples with Pro/Pro (10/20; 50%) genotype were more likely to harbor mutation than samples with Arg/Arg genotype (5/16; 31.3%) (p = 0.289) (Table 2). Therefore we proceeded to determine whether Pro72 allele was more frequently mutated by analyzing samples heterozygous for Pro72Arg polymorphism. A single PCR product that included the mutation as well as the polymorphism was generated and cloned into a suitable plasmid vector and sequenced to determine whether the mutation was present in the Pro72 or the Arg72 allele. As shown in Figure 2, the mutation was preferentially located in the Pro72 allele (p = 0.0001) (Table 2), thus supporting the result obtained for samples homozygous for codon 72 and enabling us to compare Pro72Arg polymorphism and p53 mutation status for all 75 ESCC samples which revealed that mutation was significantly associated with Pro72 allele (p = 0.0018) (Table 2).

**Table 2:** Comparative analysis of p53 mutation and Pro72Arg polymorphism status in SCCOT and ESCC samples.

Codon 72 genotype	P53 mutation status		Significance
	Present	Absent	
**SCCOT all samples (Stratified by genotype)**			
Pro/Pro (22)	07	15	0.917^a^
Pro/Arg (27)	08	19	
Arg/Arg (12)	03	09	
**ESCC all samples (Stratified by genotype)**			
Pro/Pro (20)	10	10	0.289^a^
Pro/Arg (46)	14	32	
Arg/Arg (16)	05	11	
**ESCC samples heterozygous for codon 72 polymorphism**			
Pro72	13	01	0.000^b^
Arg72	01	13	
**ESCC all samples (Stratified by allele)**			
Pro72	23	63	0.0018^b^
Arg72	6	72	

a from χ^2^ test.

b from Fisher's exact test.

Interestingly, mutations expected to cause p53 truncation (nonsense and frameshift) were exclusively identified in the Pro72 allele (8/8, 100%) in ESCC samples. Though, one deletion and one complex mutation (of total six mutations) were identified in Arg72 allele, both were not expected to alter reading frame and thus may not cause protein truncation. Only 16.67% of *TP53* mutations in SCCOT [26] (as compared to 27.59% in ESCC) were expected to result in p53 truncation, further suggesting differences in biology of SCCOT and ESCC with respect to p53 function.

## Discussion

Several studies conducted on mixture of Head and Neck squamous cell carcinoma (HNSCC) samples did not detect any significant association with *TP53* codon 72 polymorphism [31]–[33] perhaps due to heterogeneity of tumor subtypes. Studies on tumors of specific HNSCC site and/or molecular subtype did however reveal association with polymorphism in different ethnicities [34]–[37]. To the best of our knowledge, this is the first specific case control study undertaken to find the association between *TP53* Pro72Arg polymorphism and SCCOT. There are conflicting reports on the association of Pro72Arg polymorphism with ESCC risk with studies reporting significant association with Pro72 [22], [23], [38], [39], Arg72 [40] or neither [41], [42]. Interestingly, the effect may vary within the same population [22], [40]. Our analysis revealed that Pro72 allele was significantly associated with SCCOT but not with ESCC, perhaps reflecting differences in biology of SCCOT and ESCC among Indians. Of note, a recent study suggested significant difference in p53 function between Arg72 and Pro72 allele. However this difference was tissue specific [43]. It was also suggested that Pro72 allele could be more efficient in causing cell cycle arrest [44] whereas Arg72 allele was shown to be more efficient in inducing apoptosis and localization to mitochondria [45]. Though it has been shown that Arg72 allele could be more efficiently targeted by HPV protein E6 [29], we did not detect any significant association in this study.

Similar to our results, another study from India also reported absence of *TP53* mutations in ESCC tumors without nuclear stabilization [46], unlike reports from other countries (54–55) perhaps indicating a feature exclusively associated with Indian population. In addition, the ESCC *TP53* somatic mutation spectrum detected in this study is similar to the observations made earlier from India [47]. Lower proportion of G:C>A:T transitions at CpG dinucleotides (attributed to spontaneous deamination of cytosine) identified in ESCC samples in this study is in line with previous studies from India [47]. Higher frequency of G:C>A:T transitions at non-CpG sites can be attributed to alkylating agents in food and environment [48]. Of note, nitrosamines in alcoholic beverages [49] and processed meat [50] are known to increase the risk of esophageal and stomach cancer, respectively. ESCC studies from Iran [51] as well as southern Brazil [52] reported an association of habit of drinking hot tea with G:C>A:T transitions.

To the best of our knowledge, this is the only study to evaluate status of *TP53* mutation in Pro/Arg heterozygotes, which is expected to provide more accurate information on extent of association since both alleles are present in the same genetic background. We observed that p53 DNA binding domain mutations were predominantly detected in Pro72 allele (compared to Arg72 allele) in ESCC whereas neither allele was preferentially mutated in SCCOT. Several studies on various cancers reported possible association of p53 DNA binding domain mutation with Arg72 allele [53], [54] while a few reported otherwise [24], [55]. Perhaps, the association may vary according to ethnicity [24]. Mutated p53 Arg72 is suggested to be more effective compared to p53 Pro72 in binding and inactivating p73, a p53 homologue that can transactivate p53 targets [56]. In addition, previous studies also showed that mutated Pro72 allele may have higher potential to inactivate p53 itself [57]. Of note, ESCC exhibits frequent inactivation of p73 through loss of heterozygosity [58], thereby perhaps explaining higher frequency of mutation in Pro72 allele causing inactivation of p53.

Interestingly, we observed protein truncating mutations associated exclusively with the Pro72 allele in ESCC. Of note, truncation mutations are expected to completely abrogate p53 function while missense mutations can be expected to retain partial/altered activity. In addition, unlike truncation mutation, missense mutations in p53 DNA binding domain may alter the ability of p53 to bind to DNA and transactivate target genes but may not affect p53 functions exclusive to other domains of protein [59]. Thus complete inactivation of Pro72 might be more tumorigenic than Arg72 in ESCC.

## Conclusion

This study has revealed effect of *TP53* Pro72 allele in increasing the risk of SCCOT and suggested that SCCOT may have biological difference with other forms of HNSCC. A unique feature of this study was determination of mutation status in samples heterozygous for p53 Pro72Arg polymorphism, which enabled us to conclude that Pro72 allele was indeed preferentially mutated in ESCC. Our results support previous observations suggesting different behavior of the p53 Pro72Arg alleles in different cancer types and ethnicities and suggest distinct molecular function of p53 Arg72 and p53 Pro72 with respect to associated mutation, in ESCC. Further studies on other cancers should be conducted to analyze the association of polymorphism and mutation. Our results can be extended to analyze the effect of polymorphism and mutation on patient survival and response to chemo/radiotherapies. Molecular and functional studies on mutant p53 in Pro72 and Arg72 background could possibly elucidate differential activity of p53. Finally, to elucidate the effect of polymorphism on mutation, studies using animal models may help in understanding the role of this polymorphism in tumorigenesis.

## Supporting Information

Figure S1 **Comparative analysis of distribution of ESCC** ***TP53*** **mutations in India with the IARC** ***TP53*** **database at nucleotide level (Panel A), DNA level (Panel B) and protein level (Panel C).** Unclassified and silent mutations reported in the database were omitted from this analysis. The p.V143_W146delinAV mutation was not included in the analysis (in Panel C) due to complexity of its effect at protein level.
(EPS)

Table S1 (DOCX)

Table S2 (DOC)

Table S3 (DOCX)

Table S4 (DOC)

Table S5 (DOCX)

Document S1 (DOCX)

Document S2 (DOCX)
