# Supplementary material for: TP53 Pro72 Allele Is Enriched in Oral Tongue Cancer and Frequently Mutated in Esophageal Cancer in India
Source: PLoS One. 2014 Dec 1;9(12):e114002. doi: 10.1371/journal.pone.0114002 (PMC4250174; doi:10.1371/journal.pone.0114002)
Supplement: Table S1 — (DOCX) [file pone.0114002.s002.docx]

**Table S1: Clinico-pathological details of SCCOT and ESCC patients**

| **Variable** | **Group** | **SCCOT** | | | | | **ESCC** | | | | |
| --- | --- | --- | --- | --- | --- | --- | --- | --- | --- | --- | --- |
|  |  | **N** | **Pro/Pro** | **Pro/Arg** | **Arg/Arg** | **p^a^** | **N** | **Pro/Pro** | **Pro/Arg** | **Arg/Arg** | **p^a^** |
|  |  |  |  |  |  |  |  |  |  |  |  |
| Age | ≤40 | 39 | 12 | 18 | 9 | 0.294 | 22 | 8 | 10 | 4 | 0.442 |
|  | 41-60 | 47 | 23 | 18 | 6 |  | 36 | 9 | 21 | 6 |  |
|  | ≥61 | 29 | 9 | 12 | 8 |  | 24 | 3 | 16 | 5 |  |
|  |  |  |  |  |  |  |  |  |  |  |  |
| Gender | Male | 76 | 28 | 35 | 13 | 0.358 | 41 | 9 | 26 | 6 | 0.514 |
|  | Female | 39 | 16 | 13 | 10 |  | 41 | 11 | 21 | 9 |  |
|  |  |  |  |  |  |  |  |  |  |  |  |
| Tobacco | Never users | 21 | 8 | 10 | 3 | 0.969 | 30 | 10 | 16 | 4 | 0.773 |
|  | Users | 54 | 20 | 25 | 9 |  | 24 | 6 | 15 | 3 |  |
|  |  |  |  |  |  |  |  |  |  |  |  |
| Alcohol | Never users | 35 | 17 | 13 | 5 | 0.742 | 41 | 13 | 23 | 5 | 0.970 |
|  | Users | 40 | 16 | 18 | 6 |  | 10 | 3 | 6 | 1 |  |
|  |  |  |  |  |  |  |  |  |  |  |  |
| Grade | Well differentiated | 99 | 40 | 40 | 19 | 0.921 | 50 | 11 | 28 | 11 | 0.071 |
|  | Moderately differentiated | 11 | 3 | 6 | 2 |  | 23 | 5 | 14 | 4 |  |
|  | Poorly differentiated | 05 | 2 | 2 | 1 |  | 07 | 5 | 2 | 0 |  |
|  |  |  |  |  |  |  |  |  |  |  |  |
| Tumor stage | T1 | 32 | 12 | 14 | 6 | 0.956 | 26 | 4 | 20 | 2 | 0.073 |
|  | T2 | 26 | 10 | 11 | 5 |  | 13 | 6 | 4 | 3 |  |
|  | T3 | 07 | 3 | 2 | 2 |  | 09 | 3 | 4 | 2 |  |
|  |  |  |  |  |  |  |  |  |  |  |  |
| Node stage | N0 | 30 | 13 | 11 | 6 | 0.889 | 25 | 10 | 6 | 9 | 0.051 |
|  | N1 | 14 | 5 | 7 | 3 |  | 17 | 2 | 12 | 3 |  |
|  | N2 | 10 | 5 | 4 | 1 |  | 10 | 2 | 5 | 3 |  |

N, Total number of samples;

^a^p value is from χ^2^ test
