# Supplementary material for: TP53 Pro72 Allele Is Enriched in Oral Tongue Cancer and Frequently Mutated in Esophageal Cancer in India
Source: PLoS One. 2014 Dec 1;9(12):e114002. doi: 10.1371/journal.pone.0114002 (PMC4250174; doi:10.1371/journal.pone.0114002)
Supplement: Table S2 — (DOC) [file pone.0114002.s003.doc]

**Table S2: Primers used in the current study**

| Primer | Sequence (5*’—*3*’*) |
| --- | --- |
| Arg+^a,b,d,e^ | TCCCCCTTGCCGTCCCAA |
| Arg-^b,d^ | CTGGTGCAGGGGCCACGC |
| Pro+^c^ | GCCAGAGGCTGCTCCCCC |
| Pro-^a,c^ | CGTGCAAGTCACAGACTT |
| Exon 5F^e^ | TCAACTCTGTCTCCTTCCTCTT |
| Exon 5R^e^ | AACCAGCCCTGTCGTCTCTC |
| Exon 6F^e^ | CTCTGATTCCTCACTGATTGCTCT |
| Exon 6R^e^ | CCACTGACAACCACCCTTAACC |
| Exon 7F^e^ | GCACTGGCCTCATCTTGG |
| Exon 7R^e^ | GGGTCAGAGGCAAGCAGA |
| Exon 8F^e^ | CTGCCTCTTGCTTCTCTTTTCCTA |
| Exon 8R^d,e^ | ATAACTGCACCCTTGGTCTCCTC |

^a^External PCR; ^b^Arg specific PCR; ^c^Pro specific PCR; ^d^Long range PCR; ^e^Sequencing Primer;
