# Supplementary material for: TP53 Pro72 Allele Is Enriched in Oral Tongue Cancer and Frequently Mutated in Esophageal Cancer in India
Source: PLoS One. 2014 Dec 1;9(12):e114002. doi: 10.1371/journal.pone.0114002 (PMC4250174; doi:10.1371/journal.pone.0114002)
Supplement: Table S3 — (DOCX) [file pone.0114002.s004.docx]

**Table S3A. Analysis of inheritance model for codon 72 polymorphism in SCCOT patients**

| **Model** | **Genotype** | **Cases (%)** | **Controls (%)** | **OR** | **95% CI** | **P value** | **AIC** | **BIC** |
| --- | --- | --- | --- | --- | --- | --- | --- | --- |
| Co-dominant | Pro/Pro | 44 (38.3%) | 26 (23.6%) | 1.00 | - | 0.05 | 311.8 | 322 |
|  | Pro/Arg | 48 (41.7%) | 53 (48.2%) | 1.87 | (1.00-3.48) |  |  |  |
|  | Arg/Arg | 23 (20%) | 31 (28.2%) | 2.28 | (1.1-4.71) |  |  |  |
|  | | | | | | | | |
| Dominant | Pro/Pro- Pro/Arg | 92 (80%) | 79 (71.8%) | 1.00 | - | 0.15 | 313.7 | 320.6 |
|  | Arg/Arg | 23 (20%) | 31 (28.2%) | 1.57 | (0.85-2.91) |  |  |  |
|  | | | | | | | | |
| Recessive | Pro/Pro | 44 (38.3%) | 26 (23.6%) | 1.00 | - | 0.017 | 310.1 | 317 |
|  | Pro/Arg- Arg/Arg | 71 (61.7%) | 84 (76.4%) | 2.00 | (1.12-3.57) |  |  |  |
|  | | | | | | | | |
| Over dominant | Pro/Pro- Arg/Arg | 67 (58.3%) | 57 (51.8%) | 1.00 | - | 0.33 | 314.9 | 321.7 |
|  | Pro/Arg | 48 (41.7%) | 53 (48.2%) | 1.30 | (0.77-2.20) |  |  |  |
|  | | | | | | | | |
| Log-additive | - | - | - | 1.53 | (1.06-2.19) | 0.02 | 310.4 | 317.2 |

P/P, Pro/Pro genotype; P/R, Pro/Arg genotype; R/R, Arg/Arg genotype, OR, Odds Ratio; AIC, Akaike information criterion; BIC, Bayesian information criterion

**Table S3B. Analysis of inheritance model for codon 72 polymorphism in ESCC patients**

| **Model** | **Genotype** | **Cases (%)** | **Controls (%)** | **OR** | **95% CI** | **P value** | **AIC** | **BIC** |
| --- | --- | --- | --- | --- | --- | --- | --- | --- |
| Co-dominant | Arg/Arg | 16 (19.5) | 31 (28.2%) | 1.00 | - | 0.36 | 266 | 275.8 |
|  | Pro/Arg | 46 (56.1%) | 53 (48.2%) | 0.59 | (0.29-1.22) |  |  |  |
|  | Pro/Pro | 20 (24.4%) | 26 (23.6%) | 0.67 | (0.29-1.55) |  |  |  |
|  | | | | | | | | |
| Dominant | Arg/Arg | 16 (19.5%) | 31 (28.2%) | 1.00 | - | 0.16 | 264.1 | 270.6 |
|  | Pro/Pro- Pro/Arg | 66 (80.5%) | 79 (71.8%) | 0.62 | (0.31-1.23) |  |  |  |
|  | | | | | | | | |
| Recessive | Pro/Arg- Arg/Arg | 62 (75.6%) | 84 (76.4%) | 1.00 | - | 0.9 | 266.1 | 272.6 |
|  | Pro/Pro | 20 (24.4%) | 26 (23.6%) | 0.96 | (0.49-1.87) |  |  |  |
|  | | | | | | | | |
| Over dominant | Pro/Pro- Arg/Arg | 36 (43.9%) | 57 (51.8%) | 1.00 | - | 0.28 | 264.9 | 271.4 |
|  | Pro/Arg | 46 (56.1%) | 53 (48.2%) | 0.73 | (0.41-1.29) |  |  |  |
|  | | | | | | | | |
| Log-additive | - | - | - | 0.82 | (0.54-1.24) | 0.35 | 265.2 | 271.7 |

P/P, Pro/Pro genotype; P/R, Pro/Arg genotype; R/R, Arg/Arg genotype, OR, Odds Ratio; AIC, Akaike information criterion; BIC, Bayesian information criterion
