# Supplementary material for: TP53 Pro72 Allele Is Enriched in Oral Tongue Cancer and Frequently Mutated in Esophageal Cancer in India
Source: PLoS One. 2014 Dec 1;9(12):e114002. doi: 10.1371/journal.pone.0114002 (PMC4250174; doi:10.1371/journal.pone.0114002)
Supplement: Table S4 — (DOC) [file pone.0114002.s005.doc]

**Table S4: *TP53*** mutations identified in ESCC samples.

| **S.No** | **Tumor** | **Exon** | **Mutation** | | **Mutation type** | | **Codon 72 Genotype** | **Mutated allele** |
| --- | --- | --- | --- | --- | --- | --- | --- | --- |
|  |  |  | **DNA** | **Protein** | **DNA** | **Protein** |  |  |
| 1 | 3357 | 5 | c.535C>T | p.H179Y | Transition | Missense | Pro/Pro | Pro |
| 2 | 3529 | 5 | c.488A>G | p.Y163C | Transition | Missense | Pro/Pro | Pro |
| 3 | 3371 | 8 | c.814G>A | p.V272M | Transition | Missense | Pro/Pro | Pro |
| 4 | 3487 | 5 | c.406C>G | p.Q136E | Transition | Missense | Pro/Pro | Pro |
| 5 | 3519 | 7 | c.742C>T | p.R248W | Transition | Missense | Pro/Pro | Pro |
| 6 | 3417 | 5 | c.473G>C | p.R158P | Transversion | Missense | Pro/Pro | Pro |
| 7 | 3585a | 5 | c.487T>A | p.Y163N | Transversion | Missense | Pro/Pro | Pro |
| 8 | 3313 | 5 | c.610G>T^a^, **^b^** | p.E204* | Transversion | Nonsense | Pro/Pro | Pro |
| 9 | 3563 | 6 | c.586C>T**^b^** | p.R196* | Transition | Nonsense | Pro/Pro | Pro |
| 10 | 3585b | 6 | c.566delC**^a,b^** | p.P190Lfs*57 | Deletion | Frameshift | Pro/Pro | Pro |
| 11 | 3369 | 8 | c.833C>T | p.P278L | Transition | Missense | Pro/Arg | Pro |
| 12 | 3523 | 5 | c.413C>T | p.A138V | Transition | Missense | Pro/Arg | Pro |
| 13 | 3345 | 6 | c.614A>G | p.Y205C | Transition | Missense | Pro/Arg | Pro |
| 14 | 3547 | 7 | c.707A>G | p.Y236C | Transition | Missense | Pro/Arg | Pro |
| 15 | 3407 | 8 | c.838A>G | p.R280G | Transition | Missense | Pro/Arg | Pro |
| 16 | 3353 | 8 | c.796G>A | p.G266R | Transition | Missense | Pro/Arg | Pro |
| 17 | 3389 | 5 | c.401T>G | p.F134C | Transversion | Missense | Pro/Arg | Pro |
| 18 | 3421 | 6 | c.568C>A | p.P190T | Transversion | Missense | Pro/Arg | Pro |
| 19 | 3375 | 5 | c.438G>A**^b^** | p.W146* | Transition | Nonsense | Pro/Arg | Pro |
| 20 | 3511b | 6 | c.610G>T^a^, **^b^** | p.E204* | Transversion | Nonsense | Pro/Arg | Pro |
| 21 | 3521 | 5 | c.566delC**^a.b^** | p.P190Lfs*57 | Deletion | Frameshift | Pro/Arg | Pro |
| 22 | 3475 | 6 | c.621_639del19**^b,c^** | p.D208Ifs*34 | Deletion | Frameshift | Pro/Arg | Pro |
| 23 | 3587 | 5 | c.454_466dupCCGCCC GGCACCC**^b^,**^c^ | p.R156Pfs*29 | Duplication | Frameshift | Pro/Arg | Pro |
| 24 | 3467 | 5 | c.454C>A | p.P152T | Transversion | Missense | Pro/Arg | Arg |
| 25 | 3479 | 5 | c.527G>A | p.C176Y | Transition | Missense | Arg/Arg | Arg |
| 26 | 3347 | 8 | c.844C>T | p.R282W | Transition | Missense | Arg/Arg | Arg |
| 27 | 3485 | 5 | c.514G>T | p.V172F | Transversion | Missense | Arg/Arg | Arg |
| 28 | 3413 | 7 | c.778_780delTCC | p.S260del | Deletion | Deletion | Arg/Arg | Arg |
| 29 | 3549 | 5 | c.428_432delTGCAG+ 437delG^c^ | p.V143­_W146 delinsAV | Deletion | Deletion | Arg/Arg | Arg |

Mutation nomenclature is based on Human Genome Variation Society (HGVS) recommendations, ^a^Recurrent mutation; ^b^Mutations resulting in protein truncation; ^c^Novel mutation
