# Supplementary material for: TP53 Pro72 Allele Is Enriched in Oral Tongue Cancer and Frequently Mutated in Esophageal Cancer in India
Source: PLoS One. 2014 Dec 1;9(12):e114002. doi: 10.1371/journal.pone.0114002 (PMC4250174; doi:10.1371/journal.pone.0114002)
Supplement: Table S5 — (DOCX) [file pone.0114002.s006.docx]

**Table S5: Association of clinico-pathological variables with *TP53* mutations in ESCC patients**

| **Variable** | **Group** | **N** | **Mutation** | | **p^a^** |
| --- | --- | --- | --- | --- | --- |
|  |  |  | **Present** | **Absent** |  |
|  |  |  |  |  |  |
| Age | ≤40 | 22 | 10 | 12 | 0.347 |
|  | 41-60 | 36 | 13 | 23 |  |
|  | ≥61 | 24 | 06 | 18 |  |
|  |  |  |  |  |  |
| Gender | Male | 41 | 17 | 24 | 0.248 |
|  | Female | 41 | 12 | 29 |  |
|  |  |  |  |  |  |
| Tobacco | Never users | 30 | 12 | 18 | 0.851 |
|  | Users | 24 | 09 | 15 |  |
|  |  |  |  |  |  |
| Alcohol | Never users | 41 | 16 | 25 | 0.597 |
|  | Users | 10 | 03 | 07 |  |
|  |  |  |  |  |  |
| Grade | Well differentiated | 50 | 17 | 33 | 0.829 |
|  | Moderately differentiated | 23 | 07 | 16 |  |
|  | Poorly differentiated | 07 | 03 | 04 |  |
|  |  |  |  |  |  |
| Tumor stage | T1 | 26 | 12 | 14 | 0.251 |
|  | T2 | 13 | 04 | 09 |  |
|  | T3 | 09 | 06 | 03 |  |
|  |  |  |  |  |  |
| Node stage | N0 | 25 | 10 | 15 | 0.997 |
|  | N1 | 17 | 07 | 10 |  |
|  | N2 | 10 | 04 | 06 |  |

N, Total number of samples;

^a^p value is from χ^2^ test
