# Supplementary material for: TP53 Pro72 Allele Is Enriched in Oral Tongue Cancer and Frequently Mutated in Esophageal Cancer in India
Source: PLoS One. 2014 Dec 1;9(12):e114002. doi: 10.1371/journal.pone.0114002 (PMC4250174; doi:10.1371/journal.pone.0114002)
Supplement: Document S1 — (DOCX) [file pone.0114002.s007.docx]

**DNA isolation**

DNA was isolated from fresh resected tumor (after confirming >70% neoplastic cellularity), normal tissues (after confirming absence of tumor infiltration) and peripheral blood using the DNeasy Kit (Qiagen, Hamburg, Germany) as per manufacturer’s protocol. Quantity and quality of DNA were assessed by measuring absorbance at 260 nM in spectrophotometer and loading on 1% agarose gel respectively.
