# Supplementary material for: TP53 Pro72 Allele Is Enriched in Oral Tongue Cancer and Frequently Mutated in Esophageal Cancer in India
Source: PLoS One. 2014 Dec 1;9(12):e114002. doi: 10.1371/journal.pone.0114002 (PMC4250174; doi:10.1371/journal.pone.0114002)
Supplement: Document S2 — (DOCX) [file pone.0114002.s008.docx]

**Description of novel mutations:**

**c.621_639del19 (p.D208Ifs*34):** Results in alteration of reading frame beginning from codon 208 and subsequently causes protein truncation after 34 amino acids.

**c.454_466dupCCGCCCGGCACCC (p.R156Pfs*29**)**:** Results in alteration of reading frame beginning from codon 156 and subsequently causes protein truncation after 29 amino acids.

**c.428_432delTGCAG+440delG (p.V143_W146delinsAV):** Results in replacement of amino acids 143-146 by Alanine and Valine in a region that forms part of β-strand which helps in stabilizing the loop –β sheet –α helix motif, an important domain in formation of p53 DNA binding surface [[1](#_ENREF_1)].

**Reference:**

1. Cho Y, Gorina S, Jeffrey PD, Pavletich NP (1994) Crystal structure of a p53 tumor suppressor-DNA complex: understanding tumorigenic mutations. Science 265: 346-355.
